# Integrated energy optimization and emulation attack mitigation technique for CRSN under Rayleigh fading channel

**DOI:** 10.1038/s41598-025-30933-2

**Published:** 2025-12-14

**Authors:** V. Abilasha, A. Karthikeyan

**Affiliations:** School of Electronics Engineering, Vellore Institute of Technology, Vellore, India

**Keywords:** Cognitive radio sensor network, PUEA, MFO, Rayleigh fading channel, Throughput, Engineering, Mathematics and computing

## Abstract

Cognitive Radio Sensor Networks (CRSNs) are envisioned to overcome spectrum scarcity by enabling opportunistic spectrum access. However, their performance is severely degraded by Primary User Emulation Attacks (PUEAs), inefficient routing, and unfair channel allocation. In this paper, we present an Energy and PUEA-Aware Algorithm for Rayleigh Fading Channels (EPA-RF) that integrates PUEA mitigation, energy efficiency, and fairness in CRSN operating over Rayleigh flat fading channels. A PUEA mechanism is introduced, in which a malicious user intelligently senses the spectrum environment and transmits signals that imitate genuine primary users to deceive secondary users and optimize spectrum utilization. The Moth Flame Optimization (MFO) based channel allocation scheme is employed to maximize throughput while ensuring fairness among secondary users. Further, to improve network longevity, the algorithm optimally adjusts cluster radius and supports multi-hop forwarding with minimized control overhead. Simulation results verify that the proposed integrated framework significantly enhances throughput, prolongs network lifetime, and mitigates the adverse effects of PUEA compared to existing approaches.

**Supplementary Information:**

The online version contains supplementary material available at 10.1038/s41598-025-30933-2.

## Introduction

Cognitive Radio Sensor Networks (CRSN) involve a novel approach to integrating Cognitive Radio (CR) technology with Wireless Sensor Networks (WSNs)^1^. Sensor nodes employ cognitive intelligence to identify and access vacant spectrum bands of Primary Users (PUs), enabling efficient spectrum utilization without disrupting legitimate PU operations. This potential enables further reliable and versatile connectivity while mitigating the energy and spectrum shortages that traditional WSNs encounter. If two nodes are within a given range of communication and share a common accessible network channel, they are regarded as neighbors in CRSN^2^. However, CR-enabled functions like adaptive routing, dynamic spectrum usage, and spectrum sensing add more communication and processing cost, making sensor nodes’ energy consumption issues harder^3^.

Clustering has been proven to be a possible method to improve the network’s energy usage. Clustering decreases redundant transmissions and increases the overall network lifetime by combining and reducing traffic on Cluster Heads (CHs)^4,5^. However, CHs nearer the sink transmit disproportionately larger amounts of data. The uniform clustering methods are constrained by the energy gap issue, which causes an earlier reduction in energy. Uneven clustering techniques balance the usage of energy among CHs by adjusting cluster sizes according to closeness to the sink^6^. Nevertheless, there are a number of drawbacks in the current uneven clustering techniques of CRSNs, including the fact that the majority are made for single-hop communication, ignore the ever-evolving nature of channel availability, and disregard control overhead during establishment and management. Consequently, energy-aware clustering algorithms developed primarily for CRSNs persist as mandatory.

CRSN have significant security issues because of their wide-ranging as well as adaptive spectrum access, especially in conjunction with energy concerns. Primary User Emulation Attacks (PUEAs) are actually one of the most devastating threats that exist. A PUEA occurs when a malevolent user mimics the signal properties of a genuine PU to trick Secondary Users (SUs) into giving up the spectrum. Previous studies have addressed PUEA detection using localization-based techniques such as Received Signal Strength (RSS), Angle of Arrival (AoA), and Time of Arrival (ToA) to estimate and verify the true position of transmitting entities^7^. Conversely, for large-scale CRSNs, these techniques are frequently not feasible. More advanced smart PUEAs (SPUEAs) exist, where attackers use PU traffic patterns to selectively transmit, making them much more disruptive and difficult to detect^8^.

Fading channel impacts, including exact attacker patterns of traffic, remain unexplored in PUEA, whereas previous publications have mostly concentrated on Additive White Gaussian Noise (AWGN) channels of operation or assumed static attacker activity. In real-world applications, fluctuating PU and attacker traffic, along with random fluctuations in wireless fading channels, additionally impede spectrum detection capabilities and lower SU throughput.

Since security flaws can increase energy consumption and lead to throughput deterioration, CRSN face a dual dilemma when energy disparity and PUEA concerns coexist. However, the majority of current research approaches both problems separately, either concentrating on PUEA identification without considering the utility effects associated with clustering and routing, or on efficient energy grouping, without taking malevolent attackers into account.

The main objective of this research is to develop a spectrum sensing strategy that can effectively detect and mitigate Primary User Emulation Attacks (PUEA) while simultaneously reducing energy consumption in Cognitive Radio Sensor Network. Unlike existing approaches that focus only on attack detection accuracy or energy efficiency independently, the proposed method integrates both aspects, ensuring secure spectrum access without compromising the network lifetime.

In the present work, an Energy and PUEA-aware algorithm for the Rayleigh fading channel (EPA-RF) have been proposed that incorporates energy-aware clustering and the effect of dynamic PUEA during Rayleigh flat fading channels. The following is an overview of the significant elements:

1. To address the energy gap issue, we develop an energy-aware clustering routing algorithm for CRSNs that simultaneously accounts for control overhead and data transfer. It also modifies cluster formation according to network conditions.
2. For optimal spectrum utilization, a Moth Flame Optimization (MFO) based channel allocation scheme is employed to maximize throughput while ensuring fairness among secondary users.
3. Using a dependent Markov chain, we create an intelligent PUEA traffic method under Rayleigh flat fading channels for the probabilities of detection and false alarm.
4. To provide insight into the conflict amongst network lifetime, security, and throughput performance, we examine how PUEA and clustering interact to affect SU throughput and energy efficiency.

The manuscript is organized as follows: Section II -Related work. Section III presents the proposed system model, Section IV gives the proposed algorithm, Section V provides the CH Selection & Cluster Formation process, Section VI presents the Data transmission process, and Section VII presents the performance analysis through simulations. Finally, Section VIII is the conclusion.

## Related work

Clustering algorithms have emerged as a potential solution to mitigate energy constraints in Cognitive Radio Sensor Networks (CRSNs). Based on their cluster formation strategies, existing algorithms can be broadly categorized into uniform and uneven clustering algorithms. Uniform clustering algorithms, such as IMOCRP^9^, rely on single-hop communication to the sink for CH selection, which limits network scalability and introduces single points of failure. Distributed algorithms like WCM-based SACR^10^, ESUCR^11^, and NSAC^12^ overcome some of these limitations by enabling local control-based cluster formation and data transmission. Nevertheless, in both single-hop and multi-hop CRSNs, uniform clustering fails to balance residual energy among nodes, often resulting in the energy gap problem.

Uneven clustering algorithms address energy imbalance by adapting cluster radii based on node location and residual energy. Subsequent algorithms, including OACUCAPTEEN^13^ and ESAUC^14^, incorporated additional factors such as the number of neighbors and available channels, aiming to optimize CH selection and cluster formation. Despite these improvements, most uneven clustering algorithms rely on fixed or partially defined parameters, lack consideration for multi-hop inter-cluster routing, and do not fully adapt to dynamic network conditions, which can limit their practical applicability in large-scale CRSN deployments. An energy balance-oriented clustering routing protocol^15^ for CRSNs to optimize cluster formation and extend network lifetime. Its limitation is that it does not account for multi-hop inter-cluster routing and relies on fixed parameters for cluster radius, limiting scalability and adaptability, and it does not consider security threats such as PUEA or dynamic spectrum attacks. Multi-hop clustering protocol for EH-CRSNs^16^ using SWIPT and imperfect spectrum sensing, but it does not address dynamic PU activity or security threats like PUEA.

Beyond energy efficiency, CRSN performance can be significantly affected by malicious activities such as Primary User Emulation Attacks. Traditional PUEA studies often assume continuous spectrum occupation by attackers, which may not reflect realistic scenarios. Smart Primary User Emulation Attacks (SPUEA)^8^, which selectively exploit the spectrum based on primary user (PU) activity and traffic patterns. In such scenarios, secondary users (SUs) will sense and transmit over Rayleigh flat fading channels while considering PU and SPUEA traffic, but energy consumption and network lifetime impacts are needed in terms of a CRSN. A metaheuristic-based cepstral sensing technique^17^ to enhance spectrum sensing accuracy, mitigate PUEAs, and prolong CRSN network lifetime. This approach applies cepstral feature extraction to differentiate PU and attacker signals, while Whale Optimization is used to refine decision thresholds. The method reduces sensing error and improves PUEA detection accuracy under an AWGN channel and is suitable for CRSN environments. The limitation is that it incurs higher computational complexity, which may not suit resource-constrained sensor nodes.

A Primary User Emulation detection technique^18^ using Extreme Machine Learning (ELM) combined with time and distance-based signal estimation. The approach classifies legitimate PU signals by comparing spatial proximity and temporal transmission behavior. The method is lightweight and suitable for real-time sensing in cognitive radio environments. However, the approach assumes stable channel characteristics and therefore performs poorly in Rayleigh fading environments. Additionally, it does not consider multi-hop CRSN energy constraints.

A spectrum sensing scheme^19^ using a Support Vector Machine (SVM) optimized by Bayesian techniques for detecting PUEA under dynamic channel usage. The method enhances classification accuracy by tuning model hyperparameters based on spectrum variability. While effective in distinguishing PU and attacker signals, the approach depends on pre-labeled training datasets. This leads to increased computational overhead, making it less suitable for energy-constrained sensor nodes in CRSNs. A Modified Double-Threshold Cooperative Sensing approach^20^ to reduce misdetection and false alarm rates during spectrum sensing. The scheme improves sensing reliability by aggregating decision reports from multiple secondary users and adjusting thresholds adaptively. Although this enhances sensing accuracy in cooperative networks, the method is sensitive to fading severity and network density. Moreover, it does not model or prevent advanced adaptive PUEAs.

A lightweight signal footprint and pattern matching technique^21^ to verify genuine PU transmissions. The approach compares observed signals with known PU behavior to detect anomalies caused by attackers. Its simplicity makes it computationally efficient and easy to deploy. However, this approach assumes stable PU signal patterns and struggles when attackers mimic channel variations. Additionally, fading effects were not considered in their evaluation.

An analytical evaluation of PUEA^22^ effects on secondary user throughput under Rayleigh fading conditions. This work highlights how attacker traffic intensity and channel fading can significantly degrade SU performance and provides important theoretical insight into the severity of PUEA in CR networks. However, it focuses only on performance analysis and does not propose any mitigation or detection mechanism. While existing clustering algorithms improve energy efficiency, and PUEA modeling enhances network security, there is a lack of integrated approaches that jointly consider energy-aware cluster formation and smart PUEA mitigation under Rayleigh fading channel conditions. Most clustering algorithms do not account for dynamic spectrum attacks, and PUEA studies often overlook network-level energy constraints. This motivates the development of novel schemes that simultaneously optimize cluster formation, energy utilization, and attack resilience in CRSNs. The comparative analysis of the existing algorithms is given in Table 1.

**Table 1:** Comparative analysis of the existing algorithms.

Author/Year	Technique	PUEA Handling	Network Model	Advantages	Limitations
A. Karimi, A. Taherpour, and D. Cabric^8^	Traffic-pattern-based attacker modeling to analyze SU throughput reduction.	Attack-impact study only (no countermeasure).	Rayleigh flat fading throughput evaluation.	Highlights the effect of attacker traffic severity.	Provides no mitigation or sensing improvement.
T. Stephan, F. Al-Turjman, S. J. K, and B. Balusamy^14^	Deep Learning-based PU/PUE differentiation and FDMA channel allocation.	DL-based pattern recognition for attacker detection.	CRSN and DL dataset under Rayleigh fading.	High classification accuracy, better channel fairness.	Higher node computation and memory.
J. Wang and C. Li^15^	Unequal clustering based on residual node energy to avoid early node failure.	No PUEA protection included.	Multi-hop CRSN deployment field model.	Long network lifetime, balanced energy distribution.	Vulnerable to PUEA-triggered false spectrum evacuation.
Abilasha V Karthikeyan A^17^	Cepstral sensing and Whale Optimization for decision tuning.	Detects PUEA using optimized cepstral sensing features.	CRSN model under Rayleigh flat fading.	High detection accuracy with reduced sensing overhead.	Does not handle routing-level energy balancing.
Sureka & Gunaseelan^18^	Extreme Machine Learning with Distance Signal Feature Estimation for PU and Attacker.	Classifies signals based on spatial distance and transmission pattern.	CRN simulation under AWGN channel.	Low complexity and suitable for real-time sensing.	Low robustness in Rayleigh fading, no energy-aware routing.
Ambhika ^19^	SVM with Bayesian Optimization to improve sensing accuracy in dynamic spectrum conditions.	ML-based PUEA detection using labeled signal patterns.	Simulated CR environment under flat fading.	High detection accuracy under dynamic loads.	High computational cost, and is not suitable for CRSN.
Madbushi ^20^	Modified Double-Threshold Sensing to reduce misdetection and false alarms.	Uses sensing consensus among multiple secondary users.	Cooperative SU network under Rayleigh fading.	Enhances sensing reliability in cooperative networks.	Sensitive to fading variance, attacker behavior is not modeled.
Kanhere ^21^	Signal footprint & pattern matching- based on PU verification.	Detects attackers by comparing signals with PU behavior.	Analytical CRN model (no fading considered).	Very light weight and easy to implement.	Fails against adaptive attackers.
Manyisa ^22^	Analytical throughput study quantifying PUEA impact on SU performance.	No mitigation, only evaluates performance degradation.	Rayleigh fading throughput analytical model.	Clearly demonstrates the severity of PUEA effects.	No defense mechanism proposed.
**EPA-RF** Proposed Work	Energy and PUEA-Aware Routing and MFO-based Fair Channel Allocation.	PUEA detection and optimized channel assignment.	CRSN multi-hop network under Rayleigh fading.	Improves throughput under attack, delays FND/LND, energy balance, and sensing accuracy.	Requires careful sensing threshold tuning in high-interference regions.

## System model

In an annular monitoring area, $\:N\:$homogeneous CRSN nodes are uniformly and randomly deployed, ensuring efficient access to the available spectrum bands allocated to P Primary Users (PUs) are also randomly distributed within the same region. The Semi-Markov method is used to mimic the actions of PUs . In particular, there are two states, *q*_*1*_ denotes ON and *q*_*2*_ denotes the OFF state, for every permitted channel. These two states have independent durations and follow an exponential distribution. The transition probability from a *q*_*2*_ state to a q_1_ state is denoted by *p*_*c*_, and the transition probability in the reverse way is denoted by *q*_*c*_.

### Network operation

**Fig. 1:**
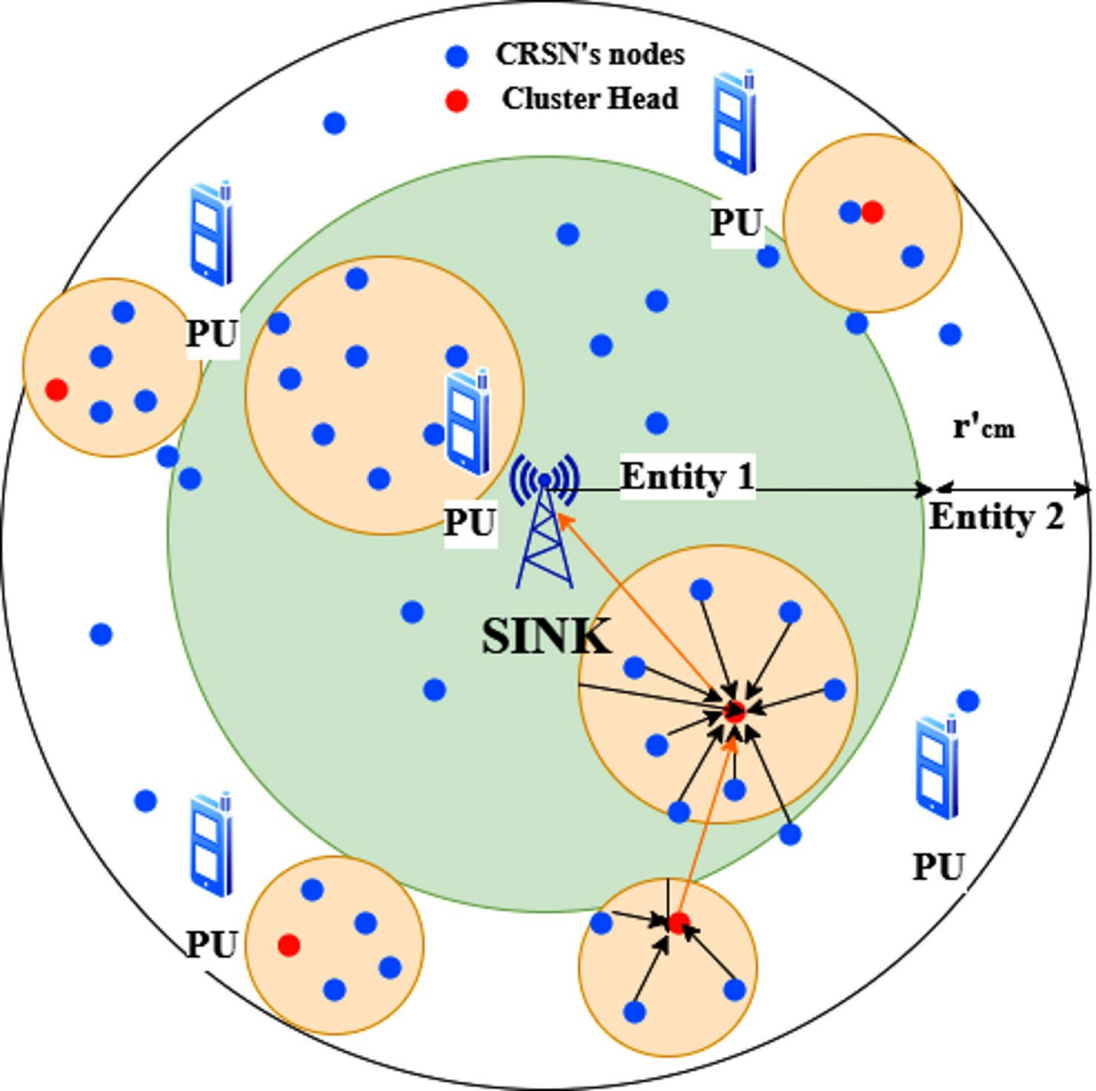
CRSN Architecture.

The network is divided into M concentric units, with entity 1 closest to the sink as shown in Fig. 1. Each node i performs spectrum sensing, cluster head (CH) selection, cluster formation, route creation, and data transmission. Every CRSN node has a distinct ID and access to its location, energy balance, available channels, and other data.

The region is separated into several annular rings (entities) encircling the sink with the same width $\:{r{\prime\:}}_{t}$ to guarantee the smallest data transmission delay and establish the cluster affiliation by considering the Euclidean distance relative to the sink.

In this case, $\:{r{\prime\:}}_{t}\:$ is the optimum communicable range of the CRSN nodes. The first entity is the one that is nearest to the sink, so that whenever the Euclidean radius between the source and sink expands, so does the entity number. Based on the Euclidean distance to the sink, *d’*_*i to sink*_*(i)*, as indicated in (5), each node of *i* can determine its entity number, $a(i) \in [1, 2, ...m,...,M]$. In this case, M represents the maximum number of CRSN entities.

1 $$\:a\left(i\right)=\frac{{d{\prime\:}}_{\text{i}\:\text{t}\text{o}\:\text{s}\text{i}\text{n}\text{k}\:\left(\text{i}\right)}}{{r{\prime\:}}_{t}\:}$$

Clusters are created by regional data exchange between nodes in a given entity m (m = 1). CMs forward the gathered data to their Cluster Head. The CH precisely integrates the incoming data and uses a long-range inter-cluster link to send the resultant information to the sink.

The use of energy for sending information is measured using the energy utilization scheme, which is frequently employed in CRSNs^9^. The consumption of energy of a transmitter that transmits *x’* bits of data to a receiver that is d meters away equals

2 $$\:{E}_{tx}\left(x{\prime\:},d{\prime\:}\right)=\left\{\begin{array}{c}x{\prime\:}\:\times\:\:\left({E}_{el}\:+\:{E}_{fs}\:{{\text{d}}^{{\prime\:}}}^{2}\right),\:\:\:\:\:\:\:if\:d{\prime\:}\:\le\:\:{\text{d}}^{{\prime\:}}0,\:\:\\\:x\:\times\:\:\left({E}_{el}\:+\:{E}_{mp}{\text{d}{\prime\:}}^{4}\right),\:\:\:\:\:\:\:otherwise\end{array}\right.$$

**Fig. 2:**
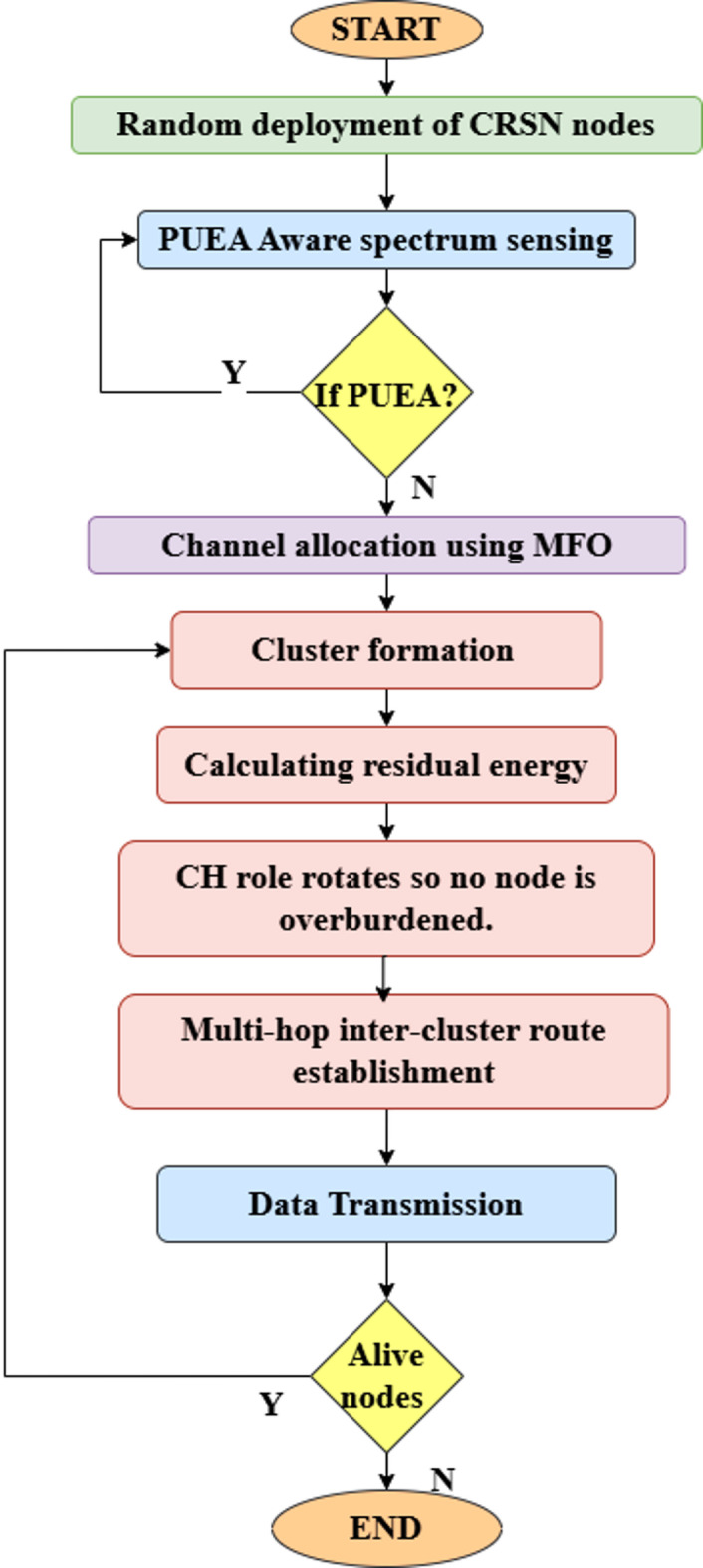
Workflow of the proposed methodology.

$\:{E}_{el}$ denotes the usage of energy of the transceiver electronics for transmitting or receiving one bit of data, *E*_*fs*_ and *E*_*mp*_ give the energy consumption values related to power amplifier circuits for the multi-path propagation model, respectively, and *d*_*0*_ *= (E*_*fs*_*/E*_*mp*_*)*^*1/2*^ is the distance threshold that establishes which path loss model should be used. The free-space loss model is used for estimating the signal distortion, which is assumed to be proportional to *d’*^*2*^ if *d < d’*_*0*_. If not, the two-ray ground reflection path loss model can be used, and d’^4^ is used to calculate the energy consumption. The workflow of the proposed work is given in Fig. 2.

### PUEA-Aware and spectrum sensing for CRSNs under Rayleigh fading

Cognitive Radio Sensor Networks (CRSNs) face dual challenges, such as limited node energy and malicious spectrum attacks by Primary User Emulation Attacks (PUEA). To tackle these, we propose an EPA-RF – Energy and PUEA-Aware Algorithm for Rayleigh Fading Channels. The approach integrates Energy balancing, uneven Clustering, and a Routing algorithm with primary user emulation attack-aware spectrum sensing, enabling prolonged network lifetime and reliable data delivery.

3 $$\:{\text{S}}_{\text{i}}=\left[{s}_{1},\:{\text{s}}_{2},\dots\:..,{\text{s}}_{\text{C}}\right],\:\:{\text{s}}_{\text{C}}=\:\left\{\begin{array}{c}1,\:\:\:\:\:\:if\:channel\:C\:is\:idle\:at\:node\:i\\\:0,\:\:\:\:otherwise\end{array}\right.$$

where *C* is the total number of licensed channels.

### PUEA-Aware spectrum sensing

**Fig. 3:**
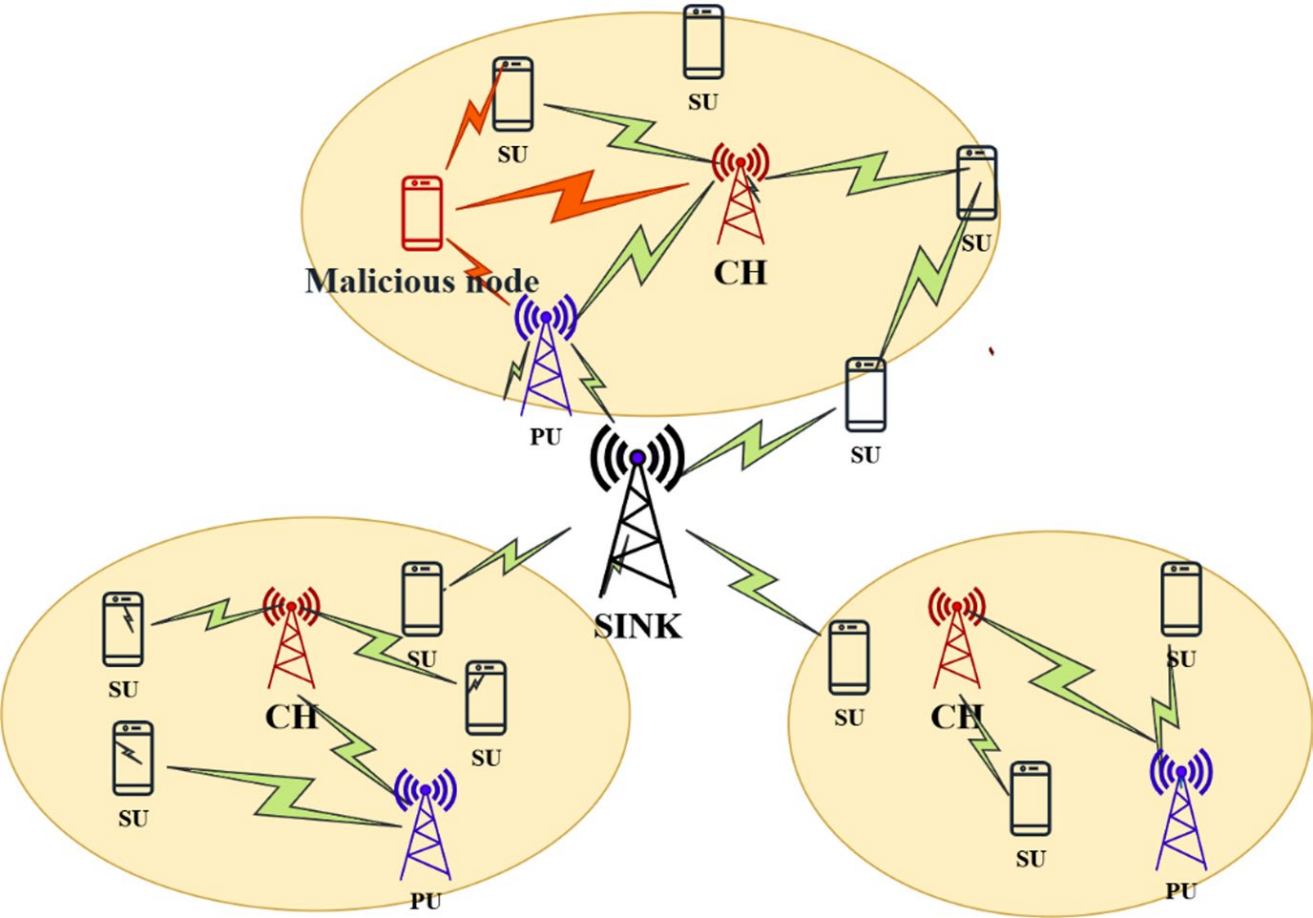
CRSN with Primary User Emulation attack.

In cognitive radio sensor networks, attackers exploit the temporary absence of primary users (PU) to gain unauthorized access to licensed channels. The CRSN architecture with Primary user emulation attack is shown in Fig. 3, and to design the dynamics of the PU and attacker (PUEA) activity, a three-state continuous-time Markov chain is used. It is assumed that every channel is a Rayleigh flat fading channel, and attackers exploit the absence of PU. A three-state continuous-time model of the PU and PUEA activity is,

- **State 0**: Channel is free, neither PU nor PUEA is present.
- **State 1**: PU is active on the channel.
- **State 2**: PUEA is active while the PU is absent.

Real primary users follow certain traffic behavior (like periodic ON and OFF activity). But attackers cannot perfectly mimic this timing and behavior. A Statistical Comparison has been made with the Secondary User compares the observed signal patterns with the known PU traffic model. If the signal timing matches the real PU, then it is PU, and if the signal timing or duration deviates, then it is a PUE attacker. SUs does not immediately leave the channel when a fake PU appears. and verify it using the traffic pattern comparison.

### Spectrum sensing considering primary user and attacker traffic

In the traffic-aware method, a Primary User Emulation Attacker (PUEA) strategically occupies the spectrum by observing the legitimate Primary User’s (PU) activity. When the PU is inactive or vacates the licensed channel, the PUEA immediately exploits this idle duration and transmits until the PU reappears.

$$\:\epsilon\:=\left\{\begin{array}{c}{\sum\:}_{k=1}^{M}{{\psi\:}_{k}}^{2},\:\:\:\:\:\:\:\:\:\:\:\:\:\:\:\:\:\:\:\:\:\:\:\:\:\:\:\:\:\:\:\:\:\:\:\:\:\:\:\:\:\:\:\:\:\:\:\:\:\:\:\:\:\:\:\:\:\:\:\:\:\:\:\:\:\:\:\:\:\:\:\:\:\:\:\:\:{\varDelta\:}_{0}\\\:{\sum\:}_{k=1}^{M}({{\varnothing\:}_{P}{m}_{k}+{\psi\:}_{k})}^{2},\:{\:\:\:\:\:\:\:\:\:\:\:\:\:\:\:\:\:\:\:\:\:\:\:\:\:\:\:\:\:\:\:\:\:\:\:\:\:\:\:\:\:\:\:\:\:\:\:\:\:\:\:\:\:\:\:\varDelta\:}_{1}\\\:{\sum\:}_{k=1}^{M}{{(\varnothing\:}_{A}{\vartheta\:}_{k}+{\psi\:}_{k})}^{2},\:{\:\:\:\:\:\:\:\:\:\:\:\:\:\:\:\:\:\:\:\:\:\:\:\:\:\:\:\:\:\:\:\:\:\:\:\:\:\:\:\:\:\:\:\:\:\:\:\:\:\:\:\:\:\:\:\:\:\varDelta\:}_{2}\\\:{\sum\:}_{k=1}^{{\delta\:}_{A}}{{(\varnothing\:}_{A}{\vartheta\:}_{k}+{\psi\:}_{k})}^{2}+{\sum\:}_{k={\delta\:}_{A}+1}^{M}{{\psi\:}_{k}}^{2},\:\:\:\:\:\:\:\:\:\:\:\:\:\:\:\:\:\:\:\:\:\:\:\:\:\:\:{\varDelta\:}_{3}\\\:{\sum\:}_{k=1}^{{\gamma\:}_{A}}{{\psi\:}_{k}}^{2}+{\sum\:}_{k={\gamma\:}_{A}+1}^{{\gamma\:}_{A}}{{(\varnothing\:}_{A}{\vartheta\:}_{k}+{\psi\:}_{k})}^{2}\:,{\:\:\:\:\:\:\:\:\:\:\:\:\:\:\:\:\:\:\:\:\:\:\:\:\:\:\varDelta\:}_{4}\\\:{\sum\:}_{k=1}^{{\delta\:}_{P}}{{(\varnothing\:}_{P}{m}_{k}+{\psi\:}_{k})}^{2}+{\sum\:}_{k={\delta\:}_{P}+1}^{M}{{(\varnothing\:}_{A}{\vartheta\:}_{k}+{\psi\:}_{k})}^{2}\:,{\:\:\:\:\:\:\varDelta\:}_{5}\\\:{\sum\:}_{k=1}^{{\gamma\:}_{P}}{{(\varnothing\:}_{A}{\vartheta\:}_{k}+{\psi\:}_{k})}^{2}+{\sum\:}_{k={\gamma\:}_{P}+1}^{M}{{(\varnothing\:}_{P}{m}_{k}+{\psi\:}_{k})}^{2}\:,\:\:\:\:\:\:\:{\varDelta\:}_{6}\end{array}\right.$$

Where M is the total number of sensing samples, $\:{m}_{k}$denotes primary user signal, $\:{\vartheta\:}_{k}$gives the attacker’s signal, $\:{\psi\:}_{k}$ provides the additive white Gaussian noise (AWGN), $\:{\varnothing\:}_{P}$and $\:{\varnothing\:}_{A}$ are the channel coefficients for PU and attacker, respectively. $\:{\gamma\:}_{A}$ and $\:{\gamma\:}_{P}$ shows the indices when the attacker or PU arrives and $\:{\delta\:}_{A},$ and $\:{\delta\:}_{P}\:a$re the sample indices when the attacker or PU departs.

### Hypothesis interpretation

- $\:{\varDelta\:}_{0}$: Both PU and PUEA are absent during the entire sensing duration.
- $\:{\varDelta\:}_{1}$: PU is continuously present; no attacker activity.
- $\:{\varDelta\:}_{2}$: PUEA is continuously present; PU remains absent.
- $\:{\varDelta\:}_{3}$: PUEA is active for the first $\:{\delta\:}_{A}$samples, then departs the channel.
- $\:{\varDelta\:}_{4}$: PUEA arrives after $\:{\gamma\:}_{A}\:$samples and remains active for the rest of the sensing period. PU stays absent.
- $\:{\varDelta\:}_{5}$: PU departs after $\:{\delta\:}_{P}$samples; PUEA utilizes the remaining time to transmit until the PU becomes active again.
- $\:{\varDelta\:}_{6}$: PUEA transmits initially, but after $\:{\gamma\:}_{P}$samples the PU returns, forcing the attacker to vacate the channel.

The transitions between these states are characterized by specific rates, which capture the probabilistic behavior of arrivals and departures of both PU and PUEA. The transition rate matrix TR, given in Eq. 4, defines the instantaneous rates at which the system moves from one state to another. These rates are determined by the activity patterns of the PU and the smart attacker, and they reflect the ON and OFF durations of their transmissions.

We model the presence of the primary user and of a PUE attacker with a binary stochastic process (0 = idle, 1 = busy). The dwell time in each state is assumed to be exponential. Specifically, the average busy durations are governed by $\:{{\upalpha\:}}_{p}$ (for the PU) and $\:{{\upalpha\:}}_{a}$ (for the attacker), and the average idle durations by $\:{{\upbeta\:}}_{p}$ and $\:{{\upbeta\:}}_{a}$, respectively. The transition rates can be,

4 $$\:TR=\left(\begin{array}{ccc}-\left({{\upalpha\:}}_{p}+{{\upalpha\:}}_{a}\right)&\:{{\upalpha\:}}_{p}&\:{{\upalpha\:}}_{a}\\\:{{\upbeta\:}}_{p}&\:-\left({{\upbeta\:}}_{p}+{\upbeta\:}\right)&\:{\upbeta\:}\\\:{{\upbeta\:}}_{a}&\:{\upalpha\:}&\:-\left({{\upbeta\:}}_{a}+{\upalpha\:}\right)\end{array}\right)$$

From the transition rates, the transition probability matrix *P*_*tr*_*(t)* can be computed using *R’(s) = (sI − Q)*^*−1*^. This matrix provides the probability that the system transitions from one state to another over time t, allowing the secondary user (SU) to anticipate the availability of channels during the sensing period. Where $\:{{\upalpha\:}}_{p}$ and $\:{{\upalpha\:}}_{a}$ are the busy channel parameters for PU and PUEA, $\:{{\upbeta\:}}_{p}$ and $\:{{\upbeta\:}}_{a}$ are the idle channel parameters for PU and PUEA. The transition probability matrix *P*_*tr*_*(t)* is given by, and it is a stochastic matrix:

5 $$\:{P}_{tr}\left(\text{t}\right)=\left({\mathcal{L}}^{-1}\left(\varvec{R}\varvec{{\prime\:}}\left(s\right)\right)\right)=\left(\begin{array}{ccc}{pm}_{00}\left(t\right)&\:{pm}_{01}\left(t\right)&\:{pm}_{02}\left(t\right)\\\:{pm}_{10}\left(t\right)&\:{pm}_{11}\left(t\right)&\:{pm}_{12}\left(t\right)\\\:{pm}_{20}\left(t\right)&\:{pm}_{21}\left(t\right)&\:{pm}_{22}\left(t\right)\end{array}\right)$$

The limiting probabilities *l*_*0*_, *l*_*1*_, and *l*_*2*_ describe the long-term steady-state behavior of the channel. These probabilities indicate the fraction of time the channel is expected to be free, occupied by the PU, or occupied by the PUEA, respectively. They are obtained by equating the rate of leaving a state to the rate of entering it, thereby ensuring equilibrium in the stochastic model.

Limiting probabilities:

6 $$\:{l}_{0}=\frac{{{{\upbeta\:}}_{p}\left({{\upbeta\:}}_{a}+{\upalpha\:}\right)+{\upbeta\:}{\upbeta\:}}_{a}}{{{\upalpha\:}}_{p}\left({{\upbeta\:}}_{a}+{\upbeta\:}+{\upalpha\:}\right)+{{\upalpha\:}}_{a}\left({{\upbeta\:}}_{p}+{\upbeta\:}+{\upalpha\:}\right)+{{\upbeta\:}}_{p}\left({{\upbeta\:}}_{a}+{\upalpha\:}\right)+{{\upbeta\:}{\upbeta\:}}_{a}}$$

7 $$\:{l}_{1}=\frac{{\upalpha\:}}{\left({{\upbeta\:}}_{p}+{\upbeta\:}+{\upalpha\:}\right)}+{v}_{0}\frac{{{\upalpha\:}}_{p}-{\upalpha\:}}{\left({{\upbeta\:}}_{p}+{\upbeta\:}+{\upalpha\:}\right)}$$

$$\:{l}_{2}=1-{l}_{0}-{l}_{1}$$.

By incorporating these probabilities, the SU can perform PUEA-aware spectrum sensing, distinguishing between legitimate PU activity and malicious PUEA activity. This awareness allows the SU to make informed decisions on channel access, minimizing interference to PUs, avoiding attacks by PUEAs, and enhancing the overall reliability and efficiency of the CRSN.

### Conditional detection probabilities under Rayleigh fading

In Rayleigh fading environments, the effectiveness of spectrum sensing is influenced by the random fluctuations of signal amplitude caused by multipath propagation. Conditional detection probabilities characterize the likelihood that a secondary user accurately detects the presence or absence of a primary user or an attacker for a given realization of the fading channel. The signal received by the secondary user comprises the faded primary user or attacker signal along with additive noise, both of which exhibit random variations governed by the Rayleigh distribution.

The conditional probability of detection reflects the probability of correctly identifying an active primary user or attacker under the current channel conditions, whereas the conditional probability of false alarm represents the likelihood of incorrectly detecting the presence of a primary user or attacker when the channel is, in fact, idle. These conditional probabilities are calculated over each sensing interval and serve as the foundation for evaluating the overall sensing performance by averaging over the statistical distribution of the fading channel. Analyzing these probabilities enables the secondary user to adjust its detection strategy dynamically, thereby improving spectrum sensing accuracy in environments with time-varying channel conditions.

For Rayleigh fading, the probability ratio test for PUEA detection can be:

8 $$\:PUEA=\frac{f\left(l\left|{\varDelta\:}_{1},{s}_{w}^{2}\right.\right)}{f(l\left|{\varDelta\:}_{0})\right.}\begin{array}{c}{\varDelta\:}_{0}\\\:\lessgtr\:\\\:{\varDelta\:}_{1}\end{array}nD$$

$$\:PUEA=\frac{{\int\:}_{0}^{{\infty\:}}\frac{1}{a}{\alpha\:}_{a}{e}^{{-\alpha\:}_{a}}a\frac{1}{\sqrt{2\pi\:{s}_{w}^{2}}}{e}^{\frac{-{l}^{2}}{2{a}^{2}{s}_{w}^{2}}}\:da}{{\int\:}_{0}^{{\infty\:}}\frac{1}{a}{\alpha\:}_{p}{e}^{{-\alpha\:}_{p}}a\frac{1}{\sqrt{2\pi\:{s}_{p}^{2}}}{e}^{\frac{-{l}^{2}}{2{a}^{2}{s}_{p}^{2}}}\:da}\:\:\:\:\:\begin{array}{c}{\varDelta\:}_{0}\\\:\lessgtr\:\\\:{\varDelta\:}_{1}\end{array}nD$$

Here, *a* is the random variable, l is the limiting probability, $\:{s}_{p}^{2}\:and\:{s}_{w}^{2}$ are the PU and PUEA signal variance, $\:{\varDelta\:}_{0}$ is the hypothesis that both PU and PUEA are absent during the entire sensing duration and $\:{\varDelta\:}_{1}$ is the hypothesis that PU is continuously present, with no attacker activity. It is interpreted as PUEA signals are discarded, and the SU uses only genuinely free channels.

- False alarm probability:

9 $$\:\begin{array}{c}{P}_{fap,TD}={\int\:}_{nD}^{{\infty\:}}f(l\left|{\varDelta\:}_{0})\right.dl\:\end{array}$$

$$\:\begin{array}{c}{P}_{fap,TD}={\int\:}_{nD}^{{\infty\:}}{\int\:}_{0}^{{\infty\:}}\frac{1}{a}{\alpha\:}_{p}{e}^{{-\alpha\:}_{p}}a\frac{1}{\sqrt{2\pi\:{s}_{p}^{2}}}{e}^{\frac{-{l}^{2}}{2{a}^{2}{s}_{p}^{2}}}\:dadl\:\end{array}$$

- Miss detection probability:

10 $$\:\begin{array}{c}{P}_{mdp,TD}={\int\:}_{-{\infty\:}}^{nD}f\left(l\left|{\varDelta\:}_{1},{s}_{w}^{2}\right.\right)dl\:\end{array}$$

$$\:\begin{array}{c}{P}_{fap,TD}={\int\:}_{-{\infty\:}}^{nD}{\int\:}_{0}^{{\infty\:}}\frac{1}{a}{\alpha\:}_{a}{e}^{{-\alpha\:}_{a}}a\frac{1}{\sqrt{2\pi\:{s}_{w}^{2}}}{e}^{\frac{-{l}^{2}}{2{a}^{2}{s}_{w}^{2}}}\:dadl\end{array}$$

## Optimal channel allocation using moth flame optimization

The Moth Flame Optimization (MFO) algorithm, introduced in^23^, is a population-based metaheuristic technique designed to efficiently explore and exploit the solution space. Initially, a population of moths is randomly generated, and their fitness values (representing the quality of their positions) are evaluated. The best-performing solution is designated as a “flame,” guiding the moths’ movement. Moths update their positions iteratively by following a spiral trajectory toward flames, continuously refining their positions until a termination condition is met. The MFO algorithm can be divided into three main steps:

### Initialization of moth population

Moths can traverse one, two, three, or even higher-dimensional search spaces. Let the moth population be represented as a matrix *B*:

11 $$\:\begin{array}{c}B=\left[\begin{array}{c}{b}_{\text{1,1}}\:{b}_{\text{1,2}}\:\dots\:\:{b}_{1,n}\\\:{b}_{\text{2,1}}\:{b}_{\text{2,2}}\:\dots\:\:{b}_{2,n}\\\:\vdots\:\:\:\:\:\:\:\:\:\: \vdots \:\:\:\:\:\ddots\:\:\:\:\:\:\: \vdots \\\:{b}_{d,1}\:{b}_{d,2}\:\dots\:\:{b}_{d,n}\end{array}\right]\:;\:F=\left[\begin{array}{c}{F}_{01}\:\\\:{F}_{02}\:\\\:\vdots\:\:\:\:\:\:\:\:\:\\\:{F}_{0n}\:\end{array}\right]\end{array}$$

Here, $\:n$ denotes the number of moths, and $\:d$ represents the dimensionality of the search space. The associated fitness values are stored in an array *F*. Flames, representing the best solutions in the population, are similarly stored in matrix *F’* with their fitness values *B*:

$$\:B=\left[\begin{array}{c}{f}_{\text{1,1}}\:{f}_{\text{1,2}}\:\dots\:\:{f}_{1,n}\\\:{f}_{\text{2,1}}\:{f}_{\text{2,2}}\:\dots\:\:{f}_{2,n}\\\:\vdots \:\:\:\:\:\: \vdots\:\:\:\:\ddots\:\:\:\:\:\:\vdots \\\:{f}_{d,1}\:{f}_{d,2}\:\dots\:\:{f}_{d,n}\end{array}\right];\:F^{\prime\:}=\left[\begin{array}{c}{f}_{01}\:\\\:{f}_{02}\:\\\: \vdots\:\:\:\\\:{f}_{0n}\:\end{array}\right]$$

### Updating moth positions

Moths update their positions according to a logarithmic spiral function, moving toward flames as follows, $\:U\left({B}_{i}^{0},\:{F}_{j}\right)={D}_{i}.{e}^{c{\prime\:}r}.\text{cos}\left(2\pi\:t\right)+{F}_{j},\:{D}_{i}=\left|{F}_{j}-{B}_{i}\right|$, where *D*_*i*_ gives the distance between a moth and its corresponding flame, *c’* is a constant, and *r* is a random number within the range [− 1,1].

### Updating the number of flames

To balance exploration and exploitation, *MF*_*O*_ reduces the number of flames over iterations, enhancing global search while avoiding premature convergence:

$$\:{MF}_{0}=rnd\left[N-K{\prime\:}.\frac{N-1}{{K{\prime\:}}_{max}}\right]$$

In this context, $\:M{F}_{0}$ refers to the present number of flames, $\:{K}^{{\prime\:}}$corresponds to the current iteration count, $\:N$ denotes the maximum number of flames, and $\:{K}_{\text{max}}^{{\prime\:}}$ represents the total number of allowable iterations.

### Optimization formulation

For channel allocation, each moth represents a candidate solution, with its position vector indicating the channel assigned to each node. Since moth positions are initially continuous, discretization is applied by rounding each element to the nearest integer. This ensures that each node is assigned to a valid channel. For example, a position vector might indicate Node 1 to Channel 2, Node 2 to Channel 1. The optimization objective is to maximize network throughput while maintaining fair channel allocation. The fitness function is defined as:

$$\:fit=\frac{{R}_{t}}{1+{P}_{co-ef}}$$

The fitness function of the moth flame optimization technique depends on the constraint.

$\:{C{\prime\:}}_{1}:{F}_{min}\le\:{F}_{NC}\le\:{F}_{max}$, where *F*_*NC*_​ is the number of nodes assigned to each channel, and *F*_*min*_​ and F_max_​ define the minimum and maximum allowable nodes per channel. *R*_*t*_ the throughput and *P*_*co−ef*_ is the penalty coefficient ​ that accounts for violations of the constraint *C’*_*1*_​, reducing the fitness of infeasible solutions. For instance, with 10 nodes and 4 channels, each channel should have between 2 and 3 nodes, ensuring equitable distribution. Each element of the vector represents the channel assigned to the corresponding node. This ensures equitable allocation; for example, with 4 channels and 10 nodes:

- Channel 1 → Nodes 2, 6.
- Channel 2 → Nodes 1, 4, 9.
- Channel 3 → Nodes 3, 7, 10.
- Channel 4 → Nodes 5, 8.

## Distributed CH selection & cluster formation

Each node computes a weight value, *Weight(i)* for CH eligibility:

12 $$\:\begin{array}{c}Weight\left(i\right)=\frac{{\text{E}\:}_{r}\left(i\right)}{{\text{E}}_{av}\left(i\right)}\:.\:{\text{C}}_{\text{u}\text{t}\text{i}}\left(i\right)\end{array}$$

*E*_*r*_*(i)* shows the Residual energy of node i and *E*_*av*_*(i)* denotes the Average energy cost to communicate with neighbors

13 $$\:\begin{array}{c}{E}_{av}\left(i\right)=\frac{\left(\left|{n}_{cnt}\left(i\right)+1\right|\right)\left({E}_{el}+{E}_{DA}\right)+{f}_{s}{d{\prime\:}}_{i\:to\:sink}^{2}{l{\prime\:}}_{1}}{\left|{n}_{cnt}\left(i\right)\right|}+\frac{\left(\left|{n}_{cnt}\left(i\right)+1\right|\right)\left({E}_{el}\right)+{f}_{s}{r}_{cm}^{{\prime\:}2}{l{\prime\:}}_{2}}{\left|{n}_{cnt}\left(i\right)\right|}\end{array}$$

14 $$\:\begin{array}{c}{C}_{uti}\left(i\right)=\frac{{\sum\:}_{j\:to\:{n}_{cnt}\left(i\right)}{\:S}_{i}{.S}_{j}}{\left|{n}_{cnt}\left(i\right)\right|.C}\end{array}$$.

*C*_*uti*_*(i)* is the Channel utilization rate, *l’*_*1*_ and *l’*_*2*_ are the Data and control packet sizes, $\:{d{\prime\:}}_{i\:to\:sink}^{2}$ is the Euclidean distance from node i to the sink and $\:{n}_{cnt}\left(i\right)$: Set of neighbors sharing at least one common channel with radius *r’*_*cm*_. Nodes with the highest weight, Weight(i), become CHs and broadcast their status. Non-CH nodes join the CH with the maximum communication weight:

15 $$\:\begin{array}{c}{weight}_{i}\left(i\right)={(S}_{{CH}_{i}}.{S}_{j}/\:{E}_{tx})={(S}_{{CH}_{i}}.{S}_{j})/\left({E}_{el}+{f}_{s}{d{\prime\:}}_{j\:to\:{CH}_{i}}^{2}\right).{l^{\prime\:}}_{1}\end{array}$$

The total transmission energy between two nodes depends on the distance and channel propagation characteristics. The energy consumed to transmit bits from *CH*_*i*_ to *CH*_*j*_ is:

16 $$\:\begin{array}{c}{E}_{t}\left({CH}_{i},{CH}_{j}\right)=\left(2{E}_{el}+{E}_{fs}{d{\prime\:}}_{{CH}_{j}to{CH}_{i}}^{2}\right).{l^{\prime\:}}_{1}\end{array}$$

Where *E*_*el*_ is the energy consumed per bit by the transmitter/receiver, *Efs* is the free-space amplifier coefficient, and *d’*^*2*^_*CHj→CHi*_ is the Euclidean distance between *CH*_*i*_ and *CH*_*j*_. Similarly, the energy for direct transmission from *CH*_*i*_ to the sink is modeled using the distance-dependent path loss:

Where *d’*_*0*_ is a threshold distance separating free-space and multipath fading regions, and *E*_*fm*_ is the multipath fading amplifier coefficient. This formulation ensures realistic modeling of energy consumption for both short- and long-distance transmissions.

17 $$\:\begin{array}{c}{E}_{t}\left({CH}_{i},sink\right)=\left\{\begin{array}{c}\left({E}_{el}+{E}_{fs}{d^{\prime\:}}_{{CH}_{j}\:to\:sink}^{2}\right).{l^{\prime\:}}_{1},\:\:d^{\prime\:}\le\:{d^{\prime\:}}_{0}\\\:\left({E}_{el}+{E}_{fm}{d^{\prime\:}}_{{CH}_{i}\:to\:sink}^{4}\right).{l^{\prime\:}}_{1},\:\:d^{\prime\:}>{d^{\prime\:}}_{0}\end{array}\right.\:\end{array}$$

### Load balancing and network lifetime

The proposed next-hop relay selection strategy guarantees that:

1. Nodes with sufficient residual energy are prioritized for relaying, avoiding early depletion of critical CHs.
2. Energy-aware distance metrics ensure that data is forwarded through paths that minimize total energy consumption rather than just the shortest distance.
3. Network partition is prevented, as nodes are not overloaded, and alternative paths are considered when the primary CH has insufficient energy.

By using multi-hop routing, energy consumption is distributed across multiple CHs, extending the operational lifetime of the network while ensuring reliable data delivery to the sink.

### Optimal cluster radius

In energy-constrained cognitive radio sensor networks, the cluster radius plays a crucial role in determining both energy consumption and network lifetime. The total energy consumed by the network in a single round can be expressed as the sum of two components:

1. Intra-unit energy, *E*_*t*_*(m)*, which accounts for the energy consumed by nodes within unit mmm during sensing, communication, and cluster management.
2. Additional energy induced in inner units, *E*_*add*_*(m)*, which represents the extra energy that inner units expend to forward data from outer units toward the sink.

The combined network energy is therefore:

18 $$\:\begin{array}{c}{E}_{t}=\sum\:_{m=1}^{M}\left({E}_{t}\left(m\right)+{E}_{add}\left(m\right)\right)\:\end{array}$$

The choice of cluster radius *r’*_*cm*_ introduces a trade-off between these two energy components:

- Increasing *r’*_*cm*_: A larger cluster radius allows a CH to serve more nodes within its entity, which increases the intra-unit energy due to longer transmissions within the cluster. However, it reduces the additional energy in inner units because fewer CHs in outer units need to forward data through the inner units.
- Decreasing *r’*_*cm*_: A smaller cluster radius reduces the intra-unit energy as CHs communicate over shorter distances, but it increases the forwarding load on inner units, raising *E*_*add*_*(m).*

To balance this trade-off, a weighted optimization is applied. By assigning a weight factor *0 < α’m ≤ 1* to the intra-unit energy, the network can minimize a weighted sum of intra-unit and additional inner-unit energy, identifying the cluster radius that optimizes energy usage and prolongs network lifetime. The optimal cluster radius is then defined as:

19 $$\:\begin{array}{c}{r}_{cm}^{*}=\text{arg}min\left\{{{\alpha\:^{\prime\:}}_{m}E}_{t}\left(m\right)+{E}_{add}\left(m\right)\right\},\:\:0<{\alpha\:^{\prime\:}}_{m}\le\:1\:\end{array}$$

This approach ensures that the network avoids energy holes in inner units while preventing excessive energy consumption in outer units, achieving a balanced and efficient cluster-based network structure.

## Data transmission process

The data transmission process is designed to be energy-efficient and reliable by combining intra-cluster aggregation with inter-cluster multi-hop forwarding. Each cluster head (CH) schedules its cluster members to transmit their sensed data in dedicated time slots to avoid collisions. The CH then aggregates the received data and forwards it toward the sink. For CHs that cannot directly communicate with the sink, next-hop relay nodes are selected based on their residual energy, proximity to the sink, and spectrum availability, ensuring that data is transmitted over available channels without interference from primary users or smart attackers. To balance energy consumption across the network, the role of CH rotates among nodes, and outer CHs share the task of forwarding data from multiple clusters. This approach effectively prolongs network lifetime, reduces energy depletion in inner clusters, and maintains reliable communication even in dynamic spectrum environments like Rayleigh fading channels.

1. **Intra-cluster**: CMs send data to CH using TDMA.
2. **Inter-cluster**: CH aggregates data and forwards via selected relay nodes.
3. **Rotation**: CH role rotates among nodes in each unit, balancing energy.
4. **Energy-Aware Relay Selection**: Ensures inner CHs act as relays only when necessary, preventing energy holes.

### Multi-hop Inter-cluster routing in energy-constrained CRSNs

In cognitive radio sensor networks (CRSNs), cluster heads (CHs) are responsible for gathering data from member nodes and forwarding it to the sink. Direct communication between CHs and the sink may be energy-expensive, especially in large-scale networks. To mitigate excessive energy consumption and prolong network lifetime, multi-hop inter-cluster routing is employed, where CHs forward aggregated data toward the sink through intermediate CHs in an energy-aware manner.

### Next-Hop relay selection

For a *CH*_*i*_ in a unit m > 1, the selection of the next-hop CH CH_j_​ is governed by an energy-aware metric:

20 $$\:\begin{array}{c}{R}_{i}\left(j\right)=\left\{\begin{array}{c}\frac{{E}_{r}\left({CH}_{i}\right).{E}_{t}{(CH}_{i},{CH}_{j})}{{E}_{t}\left({CH}_{i},sink\right)},\:\:\:\:if\:{S}_{{CH}_{i}}.{S}_{{CH}_{j}}>0\\\:0,\:otherwise\end{array}\right.\end{array}$$

Where:

- *E*_*r*_ *(CH*_*i*_*)* is the residual energy of *CH*_*i*_, ensuring that nodes with higher remaining energy are preferred.
- *E*_*t*_ *(CH*_*i*_, *CH*_*j*_*)* represents the energy required to transmit data from *CH*_*i*_ to candidate *CH*_*j*_.
- *E*_*t*_ *(CH*_*i*_, *sink)* is the energy required for direct transmission to the sink.
- $\:{S}_{{CH}_{i}}.{S}_{{CH}_{j}}$​​ indicates the eligibility of *CH*_*i*_ for forwarding (e.g., based on remaining energy threshold).

This metric balances the energy load among CHs and prevents rapid depletion of heavily used nodes, which is critical for avoiding network partition.

## Numerical results and discussions

Applying EPA-RF to entities to confirm its functionality, it calculates each entity’s cluster radius by modifying the nodes’ remaining energy across various entities while maintaining node lifetime. Simulation tests have demonstrated that EPA-RF is capable of achieving excellent performance, and Table 2 shows the CRSN configuration parameters for these simulation scenarios.

**Table 2:** Simulation Parameters.

Parameter	Value
Duration of the frame	T = 20 to 60(ms)
Time of transmission	T-τ = 10 to 50 (ms)
No. of samples collected from the channel	L = 40 to 50
Samples collected from the channel in the frame duration	(J = 65)
SNR	γ_s_ = −1 dB
Radio propagation channel	Rayleigh flat fading
User’s activity model	q1/q2 random process

### Network lifetime

**Fig. 4:**
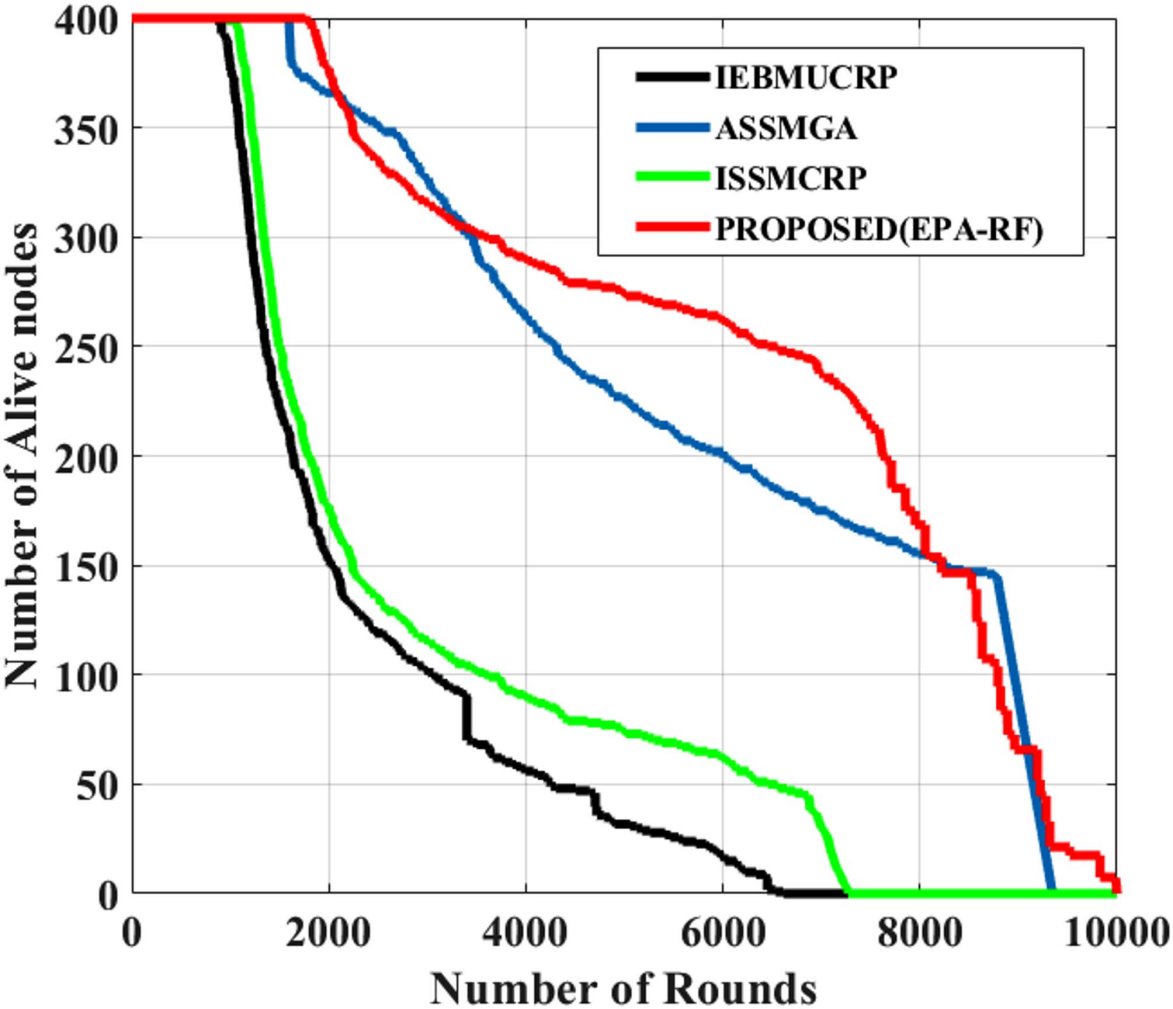
Network lifetime.

The number of surviving nodes in multi-hop CRSNs is plotted against the round number in Fig. 4. The first node death concerning EPA-RF occurs around 1767, which is much later than that of IEBMUCRP-848, ISSMCRP-966, and ASSMGA-1579. The Last node death concerning EPA-RF occurs around 9959, which is higher than that of IEBMUCRP-848, ISSMCRP-7273, but ASSMGA-9347; only a few nodes are alive. The primary motive is that EPA-RF reduces energy usage in route selection, cluster assembly, and CH selection to increase node lifespan. CRSN Nodes of entity 1 are grouped independently during CHs selection, with no control on energy use or information sharing, and the corresponding values from the plot are shown in Table 3.

**Table 3:** Network Lifetime.

Algorithms	First node death	Half node death	Last node death
**IEBMUCRP**	848	1643	6614
**ISSMCRP**	966	1788	7273
**ASSMGA**	1579	6006	9347
**PROPOSED** **(EPA-RF)**	**1767**	**7624**	**9959**

### Residual energy

**Fig. 5:**
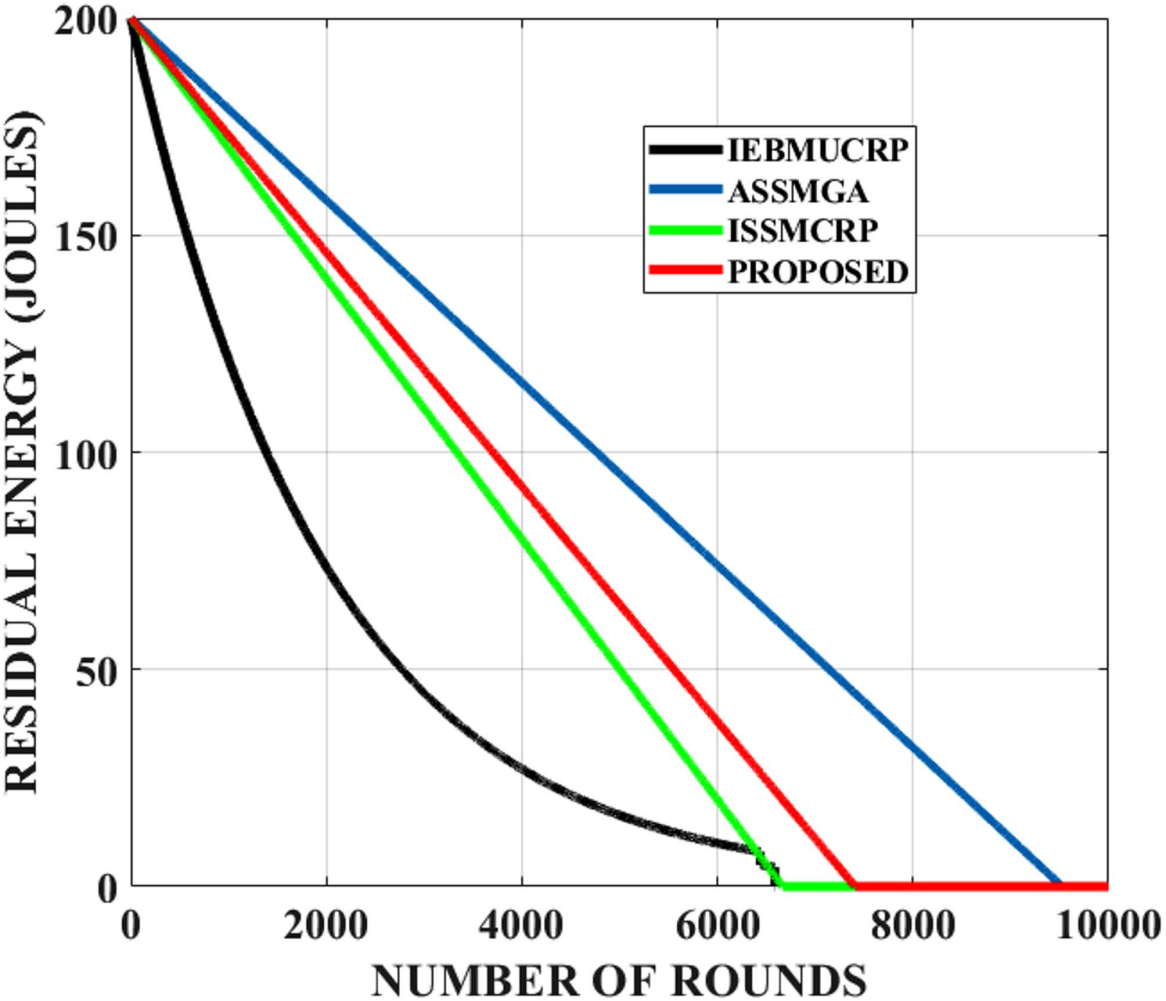
Residual energy of the network.

This metric is crucial for evaluating the network’s overall health, longevity, and performance. The residual energy needed for several algorithms, such as IEBMUCRP, ISSMCRP, and ASSMGA, as well as the suggested EPA-RF for 400 nodes, is displayed in Fig. 5. A prolonged network operation and lower energy consumption per round are suggested by the EPA-RF curve’s slower descent than the others. The network’s residual energy refers to the total remaining energy of all sensor nodes in the network after operating for a specified duration.

### Simulation and analysis in the presence of primary user emulation attack

This section examines how the performance of the SU’s networks’ spectrum sensing and throughput is affected by a number of variables, including the PU and PUEA traffic, when the PUEA is present. Furthermore, further simulations are provided to confirm the precision of the results obtained for fading channels.

### Impact of primary user and emulation attack traffic on spectrum sensing performance

**Fig. 6:**
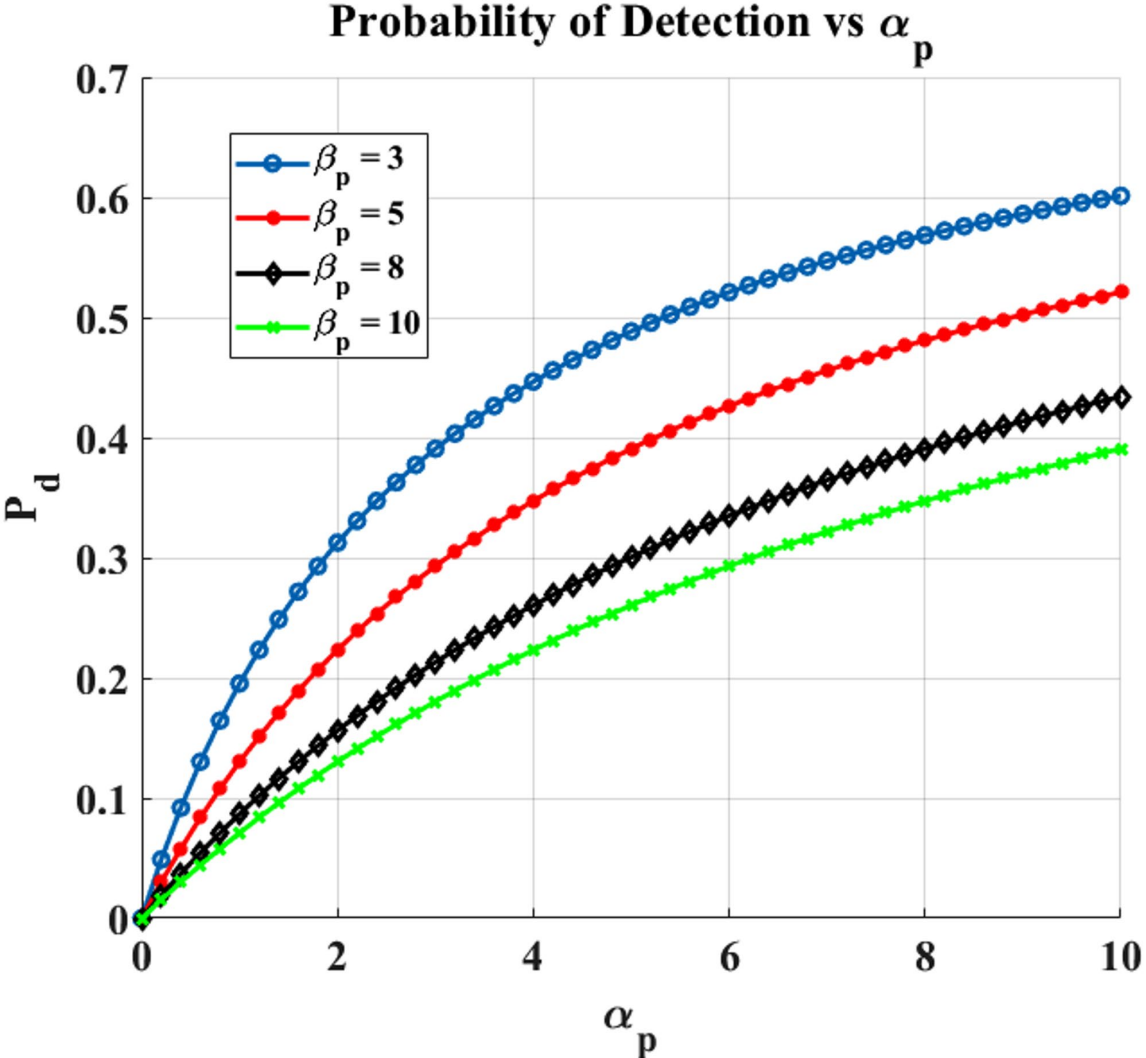
$\:\varvec{\upalpha\:}$_p_ (busy channel parameter of PU) vs. P_d_ (Probability of detection).

With varying$\:{\:{\upbeta\:}}_{p}$ Fig. 6 shows the likelihood of PU detection based on $\:{\upalpha\:}$_p_. As the likelihood of detecting PU existence rises, it is evident that P_d_ rises in parallel with $\:{\upalpha\:}$_p_. Additionally, $\:{\upalpha\:}$_p_= 0 indicates that the PU is not present in the channel, and P_d_ = 0.

**Fig. 7:**
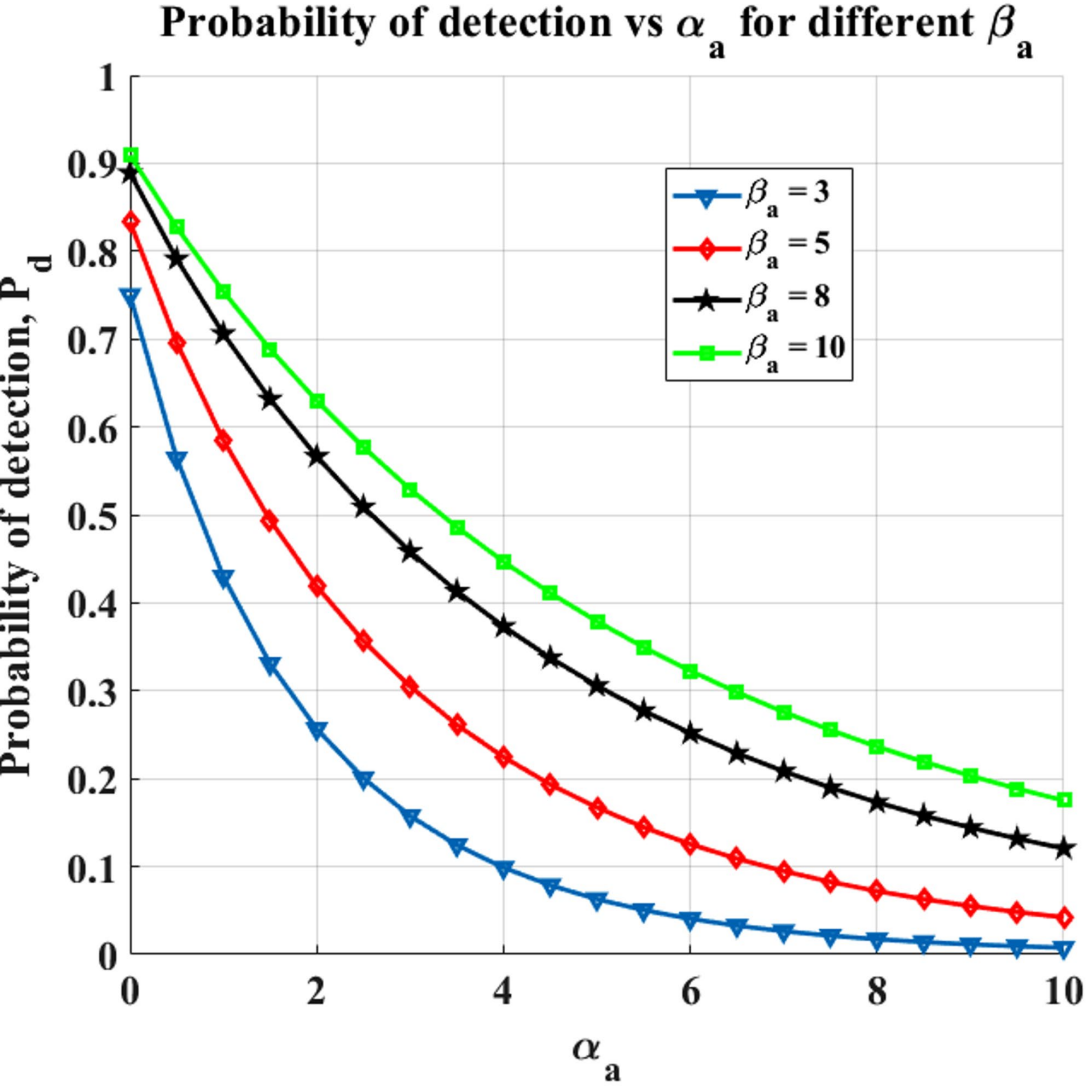
$\:\varvec{\upalpha\:}$_a_ (busy channel parameter of PUEA) vs. P_d_ (Probability of detection).

**Table 4:** Probability of detection for the Rayleigh fading Channel.

Probability of Detection for the Rayleigh Fading Channel									
$\:\varvec{\upalpha\:}$_**p**_ **vs. P**_**d**_	For different values of $\:{{\upbeta\:}}_{p}$				$\:\varvec{\upalpha\:}$_**a**_ **vs. P**_**d**_	For different values of $\:{{\upbeta\:}}_{a}$			
	3	5	8	10		3	5	8	10
$\:\varvec{\upalpha\:}_p=1$	0.19	0.13	0.08	0.07	$\:\varvec{\upalpha\:}_a=$ **1**	0.42	0.58	0.70	0.75
$\:\varvec{\upalpha\:}_p=5$	0.48	0.39	0.30	0.26	$\:\varvec{\upalpha\:}_a=5$	0.06	0.16	0.30	0.37
$\:\varvec{\upalpha\:}_p=10$	0.60	0.52	0.43	0.39	$\:\varvec{\upalpha\:}_a=10$	0.007	0.04	0.12	0.17

The chance of PU identification based on $\:{\upalpha\:}$_a_ for various $\:{{\upbeta\:}}_{a}$ values in the suggested code are shown in Fig. 7. Since the PUEA factor is present in the spectrum for a longer period of time when the value of $\:{\upalpha\:}$_a_ is higher, the likelihood of detecting PU is decreased. Furthermore, the probability of PU detection will rise as the value of $\:{{\upbeta\:}}_{a}$ increases, as this corresponds to a faster rate of PUEA departure. Table 4 provides the Probability of Detection for the Rayleigh Fading Channel for different values of $\:{{\upbeta\:}}_{p}$ and $\:{{\upbeta\:}}_{a}$.

### Throughput analysis of secondary networks under primary and emulation attack traffic conditions

**Fig. 8:**
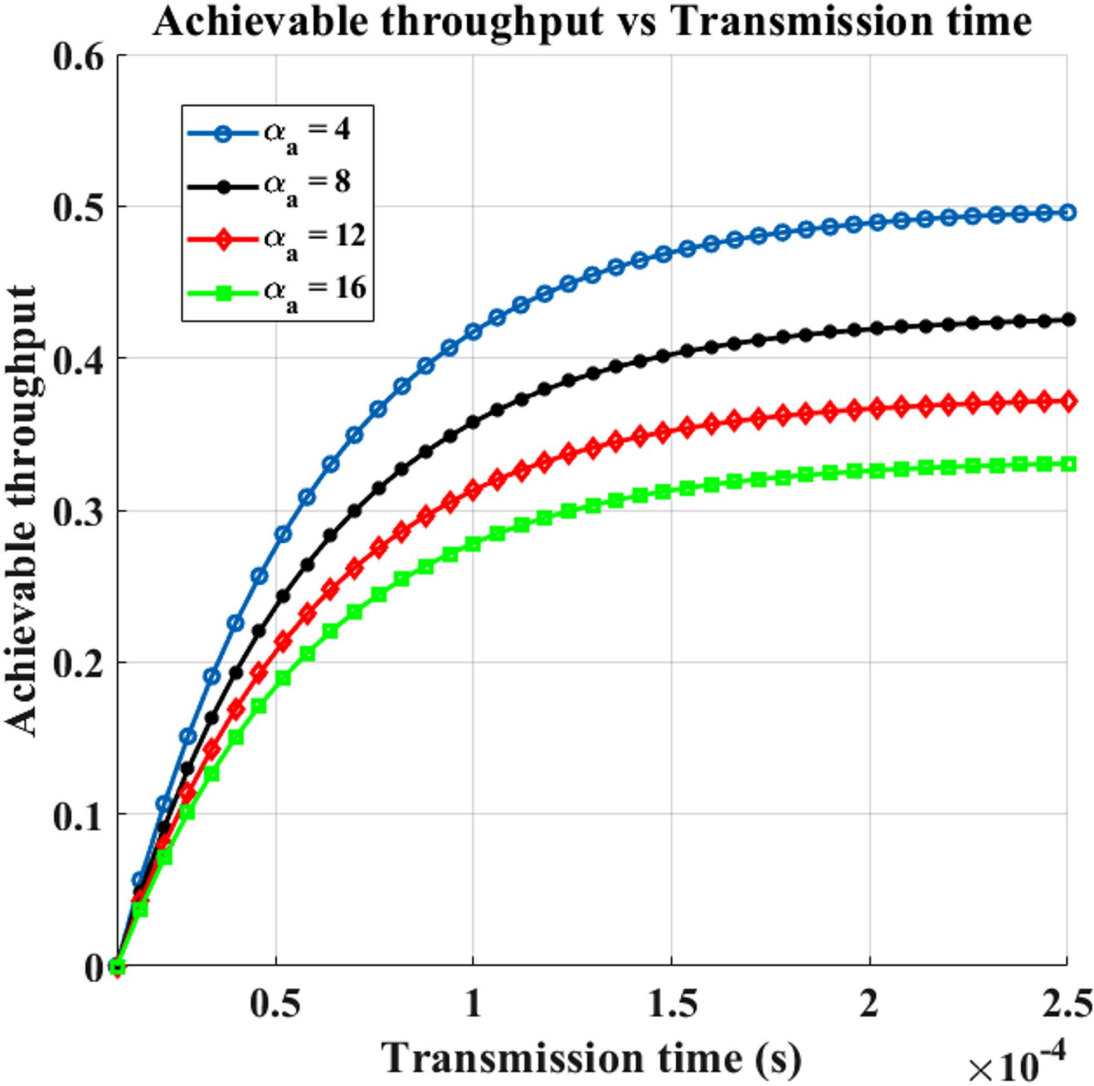
Transmission Time vs. Achievable Throughput for varying $\:{\upalpha\:}$_p_.

The mean attainable throughput versus transmission time for various PUEA traffic intensities is displayed in Fig. 8. The transition matrix *P*_*tr*_*(t)* elements are computed, describing $\:{\upalpha\:}$_p_ = 30, $\:{{\upbeta\:}}_{p}$= $\:{{\upbeta\:}}_{a}$ = $\:{\upalpha\:}$ = $\:{\upbeta\:}$ = 5, and the dependent Markov model with respect to PU and attacker activity is employed in this figure. We may derive the transition components of the matrix for additional figures in a similar manner. The network’s possible throughput rises as the transmission duration in the spectrum sensing frame structure increases. This is because data can travel across the channel for longer periods of time. It is evident that a higher $\:{\upalpha\:}$_a_, or the mean of the PUEA existence within the spectrum, will result in a lower attainable network throughput. This is because a longer length of PUEA in the spectrum increases the likelihood that SUs will wrongly identify PUEA rather than the PU. As a result, SUs will refrain from transmitting information, which lowers the possible network throughput.

**Fig. 9:**
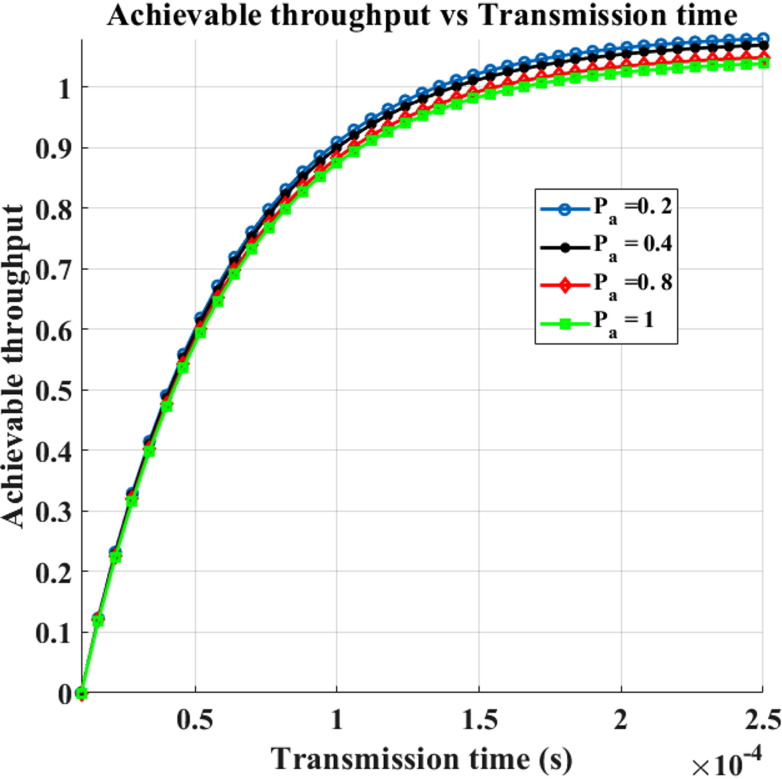
Transmission Time vs. Achievable Throughput for varying $\:\varvec{P}$_a_.

**Table 5:** Throughput for the Rayleigh fading Channel.

Time *(10^− 4^)	Throughput for the Rayleigh Fading Channel							
	$\:{\alpha\:}_{a}=4$	$\:{\alpha\:}_{a}=8$	$\:{\alpha\:}_{a}=12$	$\:{\alpha\:}_{a}=16$	$\:{P}_{a}=0.2$	$\:{P}_{a}=0.4$	$\:{P}_{a}=0.8$	$\:{P}_{a}=1$
**0.5**	**0.28**	**0.24**	**0.21**	**0.18**	**0.618**	**0.612**	**0.60**	**0.59**
**1.5**	**0.46**	**0.40**	**0.35**	**0.31**	**1.02**	**1.01**	**0.99**	**0.98**
**2.5**	**0.49**	**0.42**	**0.37**	**0.33**	**1.08**	**1.06**	**1.04**	**1.03**

Figure 9 illustrates the impact of the PUEA on the received SNR and the network throughput of the secondary user (SU) for different transmission durations. It is observed that as the SNR of the PUEA transmitter increases, the achievable throughput of the SU network decreases. In actuality, a stronger disruptive signal in the CRSN results from an increase in the acquired SNR associated with the PUEA transmitter, which raises the likelihood that SUs will recognize PUEA rather than PU. Table 5 provides the Throughput for the Rayleigh Fading Channel.

### Performance evaluation of secondary user throughput under Rayleigh and AWGN fading channels

**Fig. 10:**
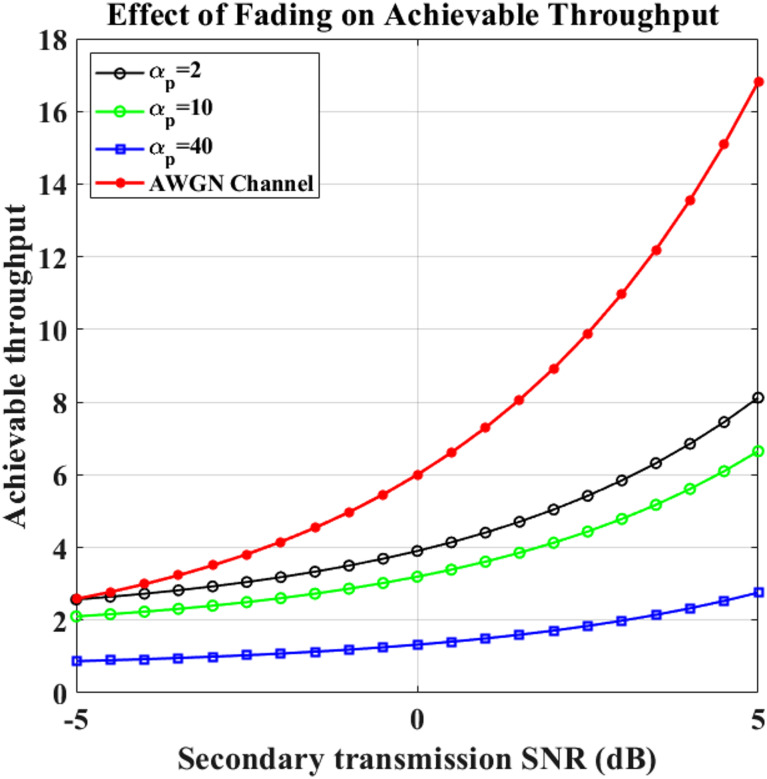
Transmission vs. Throughput with varying  $\:{\upalpha\:}_p$.

**Fig. 11:**
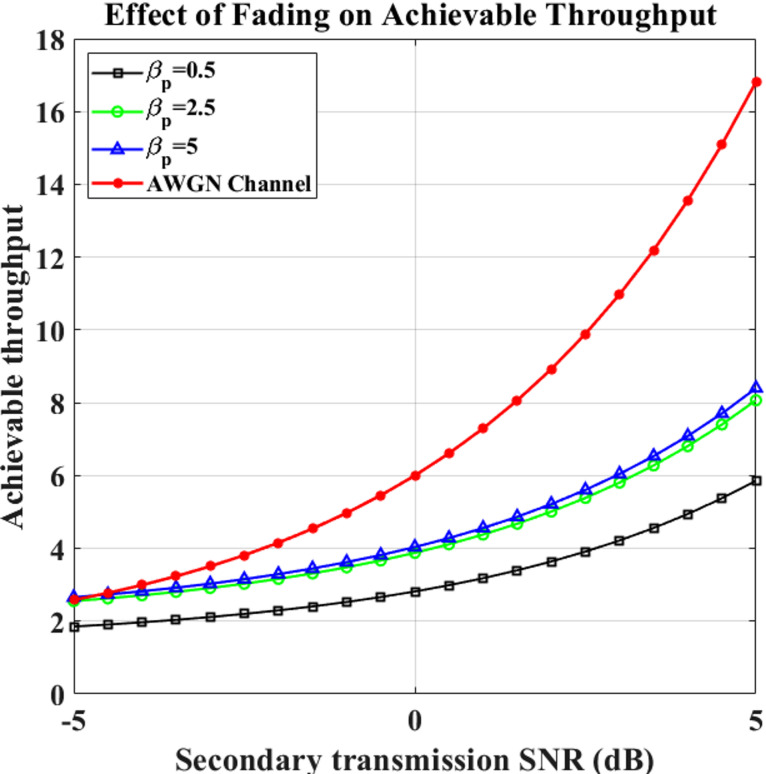
Transmission vs. Throughput with varying  $\:{{\upbeta\:}}_{p}$.

A comparison with the Rayleigh channel versus AWGN in Figs. 10 and 11 is shown, the attainable throughput of the SU’s network against the secondary transmit SNR for various values of $\:{\upalpha\:}$_p_, $\:{{\upbeta\:}}_{p}$, and $\:{\upalpha\:}$_hp_. The negative impact of higher channel average power (1/$\:{\upalpha\:}$_hp_) upon the SU’s average throughput is shown in these figures. It is clear that as the SU receiver is subjected to greater disruption from the PU source, the SU throughput decreases due to an increase in the channel average power generated by the transmission connection.

**Table 6:** Throughput for the AWGN channel and Rayleigh fading Channel.

SNR (dB)	Throughput for the Rayleigh Fading Channel						Throughput for AWGN channel
	$\:{\upalpha\:}_p=2$	$\:{\upalpha\:}_p=10$	$\:{\upalpha\:}_p=40$	$\:{{\upbeta\:}}_{p}=0.5$	$\:{{\upbeta\:}}_{p}=2.5$	$\:{{\upbeta\:}}_{p}=5$	
**−5**	**2.568**	**2.103**	**0.8722**	**1.853**	**2.552**	**2.656**	**2.581**
**0**	**3.902**	**3.195**	**1.325**	**2.815**	**3.877**	**4.035**	**6**
**5**	**8.122**	**6.649**	**2.758**	**5.859**	**8.069**	**8.398**	**16.81**

Higher values of $\:{\upalpha\:}$_p_ in the Rayleigh fading channel result in a higher possible throughput. The SU’s network throughput decreases less when the level of $\:{{\upbeta\:}}_{p}$ increases since it correlates with a larger rate of PU departure. The SU’s network throughput decreases less when the level of $\:{{\upbeta\:}}_{p}$ increases since it correlates to a higher rate of PU departure. Figure 11 shows that the Rayleigh channel with $\:{{\upbeta\:}}_{p}$ = 5 has the lowest possible throughput. Table 6 shows the Throughput analysis of the AWGN channel and Rayleigh Fading Channel.

**Fig. 12:**
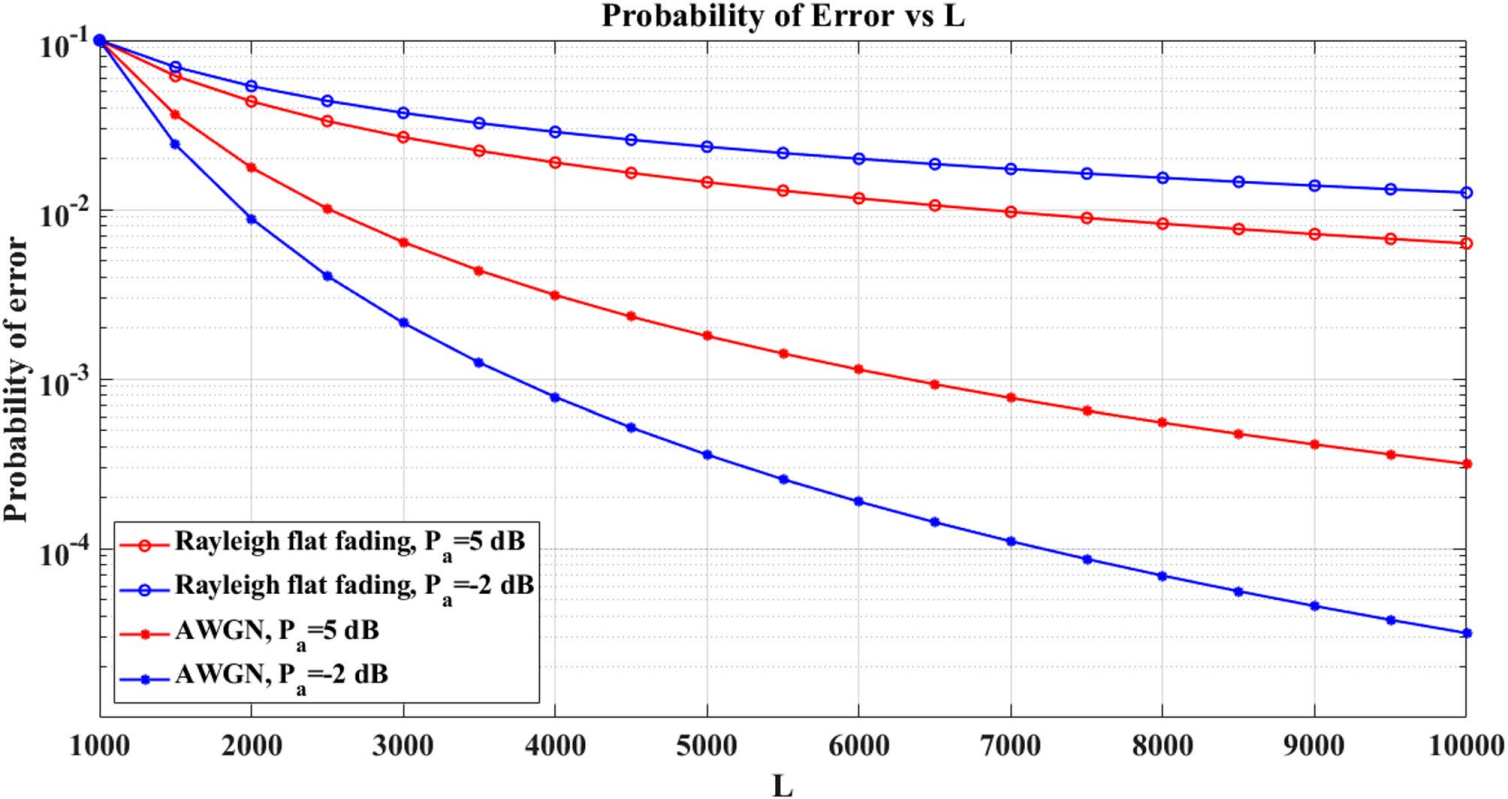
Probability of error vs. L.

We use Monte-Carlo simulation to examine the suggested identification detector for separating the PUEA and PU for various channels and PUEA SNR values in Fig. 12. Since the attacker comes with greater strength and the proposed method gathers samples from the channel with higher energy, it is evident that the likelihood of miss detection lowers as the PUEA’s SNR increases. Additionally, the technique performs better in the AWGN environment, particularly as the quantity of channel samples collected rises.

**Fig. 13:**
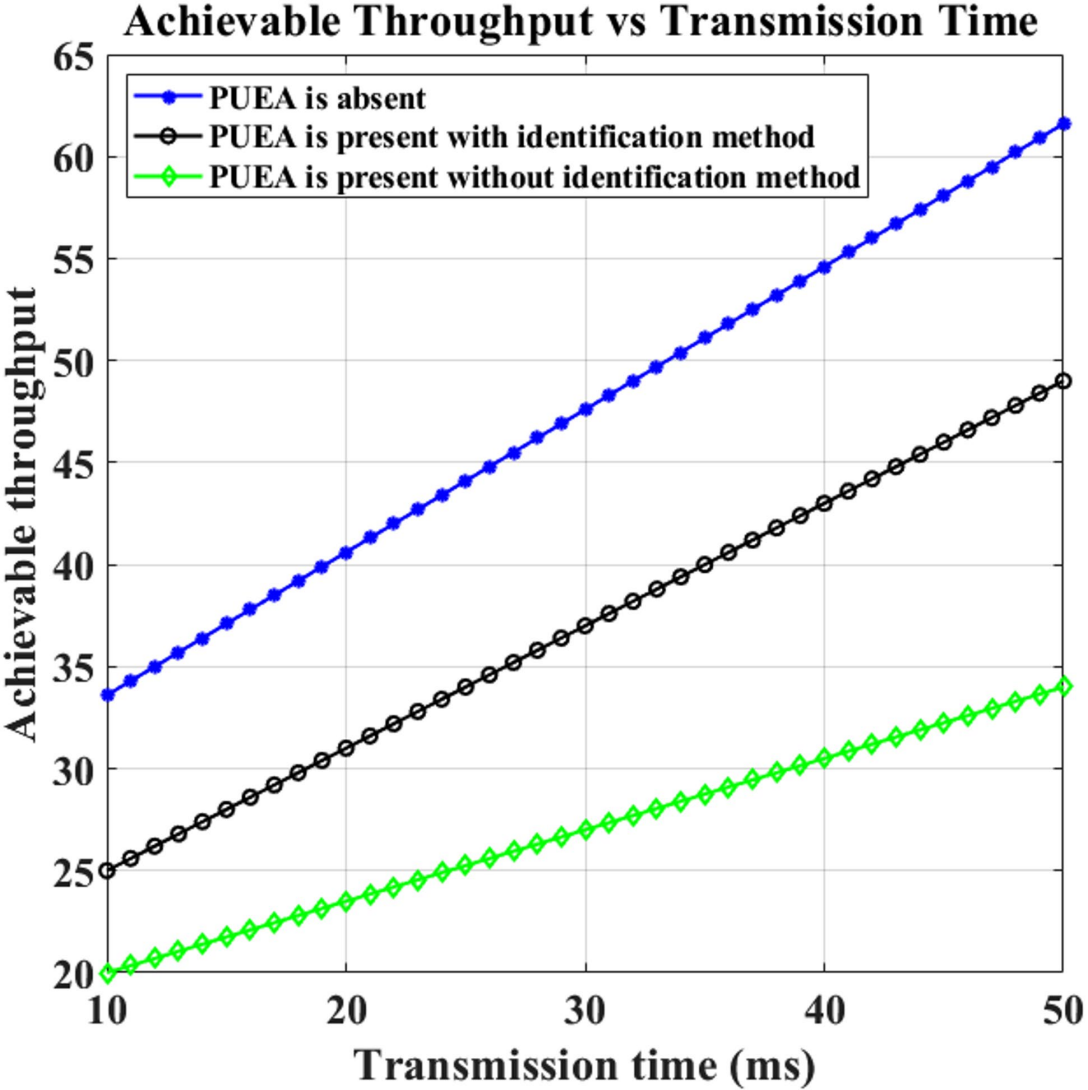
Transmission time vs. Achievable Throughput.

Lastly, a comparison of the network’s possible throughput with and without PUEA is shown in Fig. 13. Some nodes that are uniformly distributed and positioned at random inside a particular field are regarded as malevolent users. The detection and transmission times are 10 (ms) as well as 10∼50 (ms), respectively, for each cognitive radio sensor node, which comprises one radio transceiver and access to channels. At least ten runs of this simulation configuration have been performed, and the averaged results and trends have been displayed. As can be shown, SUs can accurately determine if a PU is present or absent while the PUEA is detected. As a result, employing this technique improves network performance.

## Conclusion

In this work, we proposed an integrated framework that simultaneously addresses spectrum sensing security, energy efficiency, and fair channel allocation in Cognitive Radio Sensor Networks (CRSNs). Through the implementation of a Primary User Emulation Attacker (PUEA) method under Rayleigh flat fading. It has been shown that improper traffic conditions could drive the throughput of secondary users toward zero, highlighting the severity of PUEAs. To mitigate the energy issue and enhance data delivery in multi-hop CRSNs, we developed the Energy and PUEA-Aware Algorithm for Rayleigh Fading Channels (EPA-RF), which dynamically derives optimal cluster radii and ensures a high packet delivery ratio and prolonged network lifetime. Compared with other algorithms, EPA-RF achieved better performance, as indicated by the first and last node deaths, respectively. In addition, to ensure spectrum efficiency, a Moth Flame Optimization (MFO)-based channel allocation scheme was introduced, achieving throughput gains. Simulation results validated the accuracy and demonstrated that the proposed system provides robust spectrum sensing, resilient throughput under malicious attacks, and energy-balanced communication with fair channel allocation.

Future work will focus on integrating fairness-aware metrics, leveraging artificial intelligence for adaptive cluster management, and leveraging simultaneous wireless information and power transfer to enhance the energy sustainability of CRSNs.

## Supplementary Information

Below is the link to the electronic supplementary material.

Supplementary Material 1

## Data Availability

The datasets used and/or analysed during the current study available from the corresponding author on reasonable request.
